# Placental DNA methylation marks are associated with maternal depressive symptoms during early pregnancy

**DOI:** 10.1016/j.ynstr.2021.100374

**Published:** 2021-07-31

**Authors:** Riikka J. Lund, Minna Kyläniemi, Nina Pettersson, Riina Kaukonen, Mikko Konki, Noora M. Scheinin, Linnea Karlsson, Hasse Karlsson, Eeva Ekholm

**Affiliations:** a FinnBrain Birth Cohort Study, Turku Brain and Mind Center, Department of Clinical Medicine, University of Turku, Turku, Finland; b Turku Bioscience Centre, University of Turku and Åbo Akademi University, Turku, Finland; c Department of Obstetrics and Gynecology, Turku University Central Hospital and University of Turku, Turku, Finland; d Centre for Population Health Research, University of Turku, Turku University Hospital, Turku, Finland; e Department of Psychiatry and Turku Brain and Mind Centre, University of Turku and Turku University Hospital, Turku, Finland; f Department of Pediatrics, University of Turku, Turku University Hospital, Turku, Finland

**Keywords:** Depressive symptoms, Pregnancy, Epigenome, DNA methylation, Placenta, Enhancer

## Abstract

Maternal depressive symptoms during pregnancy are a significant risk factor for adverse developmental and health outcomes of the offspring. The molecular mechanisms mediating the long-term effects of this exposure are not well understood. Previous studies have found association between prenatal exposure to maternal psychological distress and placental DNA methylation of candidate genes, which can influence placental barrier function and development of the fetus. Our objective in this study was to determine epigenome wide association of maternal depressive symptoms in early pregnancy with the placental DNA methylation. For this purpose we examined DNA methylomes of 92 placental samples by using reduced representation bisulfite sequencing. The placental samples were collected after deliveries of 39 girls and 59 boys, whose mothers had Edinburgh Postnatal Depression Score ranging from 0 to 19 at gestational week 14. According to our results maternal depressive symptoms are associated with DNA methylation of 2833 CpG sites, which are particularly over-represented in genic enhancers. The genes overlapping or nearest to these sites are functionally enriched for development of neurons and show expression enrichment in several regions of developing brain. The genomic regions harboring the DNA methylation marks are enriched for single nucleotide polymorphisms associated with mental disease trait class. Potential cellular signaling cascades mediating the effects include inflammatory and hormonal pathways. As a conclusion our results suggest that maternal depressive symptoms during early pregnancy are associated with DNA methylation marks in placenta in genes, which are important for the development and long-term health of the brain. Whether similar marks can be detected in exposed children remains to be elucidated in further studies.

## 1 Introduction

Placenta has an important function in protecting the developing fetus from harmful exposures during pregnancy. However, maternal stress, such as depressive symptoms or elevated levels of glucocorticoids during pregnancy can compromise this barrier function and increase the risk of adverse developmental and health outcomes (Moisiadis and Matthews, 2014a, 2014b). Depressive symptoms during pregnancy are common with a prevalence of 4–20% (Smith et al., 2020) and have been associated for example, with fetal hyperactivity and central adiposity (Dieter et al., 2001; Ertel et al., 2010; Gentile, 2017), recurrent respiratory infections (Korhonen et al., 2019), and motor, cognitive, language, adaptability and social-emotional outcomes (Rogers et al., 2020). Even mild maternal symptoms have been associated with emotional problems in early childhood (Pietikäinen et al., 2020). In adolescence and adulthood, increased risk of psychotic experiences and depression has been reported (Gentile, 2017; Korhonen et al., 2012; Smith et al., 2020; Srinivasan et al., 2020; Su et al., 2020; Tirumalaraju et al., 2020). On the other hand, optimal stress exposure during development may also support adaptation and resilience of the individual to stressful environment. The outcome is influenced by complex interaction of genetic and environmental factors, gender of the offspring and timing of exposure (Bale and Epperson, 2015; Sutherland and Brunwasser, 2018).

The molecular mechanisms explaining these associations are not well understood. Epigenetic regulation, such as DNA methylation, has been suggested as one of the key mediators of the effects. Epigenetic regulation is essential for proper development and function of cells and tissues, however, it is also sensitive and vulnerable to environmental influence in particular during early development. For instance, increased exposure to glucocorticoids, perceived stress, maternal depression or anxiety during pregnancy can compromise placental barrier function by affecting epigenetic status and expression of a gene encoding for HSD11B2 enzyme in placenta, which protects the fetus by converting excess levels of stress hormone cortisol to inactive cortisone (Moisiadis and Matthews, 2014b; Monk et al., 2016; O'Donnell et al., 2012; Stroud et al., 2016). Similarly, placental DNA methylation status of the glucocorticoid receptor gene, *NR3C1*, which binds cortisol, has been associated with prenatal exposure to maternal depressive symptoms and weaker self-regulation, lethargy and hypotonia of infants (Conradt et al., 2013). Nominally significant higher expression of *HTR1A* and *NPY2R* genes in the placenta of depressed mothers has also been reported (Edvinsson et al., 2019).

Our objective in this study was to determine epigenome-wide association between placental DNA methylation and maternal depressive symptoms and to gain insights into the potential functional significance of such DNA methylation marks, if identified. As DNA methylation marks can be influenced by timing of the exposure, our particular focus in this study was on the exposure measured during early pregnancy, at gestational week 14, which was the first cohort assessment in the FinnBrain Birth Cohort study (Karlsson et al., 2018). DNA methylation, which is influenced by both genetic and environmental factors, may regulate genes that are important for placental function and early development. Previous studies have examined associations between maternal psychological distress and DNA methylation status of candidate genes, such as *NR3C1* and *HSD11B2* in placenta (Ciesielski et al., 2015; Conradt et al., 2013; Monk et al., 2016). However, to our knowledge, genome-wide DNA methylome profiles have not been reported.

## 2 Materials and methods

### 2.1 Cohort and tissue description

The study sample was identified from a population based FinnBrain Birth Cohort Study (www.finnbrain.fi) including a total of 3808 mothers (Karlsson et al., 2018). The study was accepted by the Ethics Committee of the Hospital District of Southwest Finland. The participants were recruited at gestational week (gwk) 12 and gave written informed consent. The subjects filled out the Edinburgh Postnatal Depression Scale (EPDS) questionnaire three times during pregnancy, at 14, 24 and 34 gwks. The EPDS (Cox et al., 1987; Nast et al., 2013) is a validated questionnaire for screening depressive symptoms during pregnancy (Rubertsson et al., 2011). It consists of 10 items scoring 0–3. EPDS was used as a sum score (range 0–30, smaller values indicating less symptoms).

Placentas were collected from 252 singleton deliveries, samples were collected from the maternal side and were cryopreserved. Data from putative variables, which may influence the DNA methylation was collected from national Finnish Medical Birth Register (FMBR) administered by the Finnish institute for health and welfare (www.thl.fi), other questionnaire data collected as part of the FinnBrain Study and electronic patient files. These included maternal age, maternal body mass index (BMI), maternal smoking, gestational diabetes, gestational age at delivery, mode of delivery, gender of the newborn and time from delivery to freezing of the placental sample. First, subjects with placental samples and either high EPDS scores (>10 at any time point during pregnancy) or low EPDS scores (0–5 at all time points) were chosen. Second, the subjects with premature birth or glucocorticoid treatment during pregnancy were excluded. The subjects with low and high EPDS scores were stratified based on delivery route, gestational diabetes, gender of the offspring, maternal body mass index and smoking. Ninety-two placentas, which had EPDS score available from the first (14 gwk) measurement were chosen for analyses based on maternal characteristics and pregnancy outcome. These participants received an EPDS score ranging from 0 to 19. Descriptive statistics of the samples is available in Table A.

### 2.2 Nucleic acid extraction and reduced representation bisulfite sequencing

Genomic DNAs were isolated from the placental samples by using Qiagen's AllPrep DNA/RNA/miRNA Universal Kit. Libraries for Reduced Representation Bisulfite Sequencing (RRBS) were prepared from 500 ng of genomic DNA as previously described (Boyle et al., 2012; Konki et al., 2019). The libraries were quantitated with Qubit by using Quant-IT® kit (Life Technologies) and quality controlled with Fragment Analyzer (Advanced Analytics). Next-generation sequencing was performed with Illumina NovaSeq 6000 platform by using 2 × 50 bp chemistry.

### 2.3 Raw data processing and detection of DNA methylation marks

The high quality of the raw data was confirmed with FastQC v0.11.8 (Andrews, 2015) and multiQC v1.7 analyses (Ewels et al., 2016). Conversion efficiency was assessed from lambda spike-in control and was detected to be over 98.7% for all samples. The raw reads were trimmed with TrimGalore! v0.6.4 and were mapped to GRCh38 reference genome with Bismark v0.22.2. No mismatches were allowed. A range of 9,523,273 - 49,702,651 aligned reads with the mapping efficiency of 67.6–78.0% were obtained for further analyses. The CpG sites with at least 10x coverage and in at least 90% samples were included in the analysis. The permille with highest coverage was removed from the analysis. After these steps 1,586,412 GpC sites were left for the analysis. Association of EPDS with the DNA methylation of each individual CpG site was examined with PQLseq v1.10 package (Sun et al., 2019). The placental DNA methylation levels and EPDS scores were included as continuous variables in the statistical model. Covariates which could potentially influence DNA methylation, including maternal age, BMI, smoking, gestational diabetes, mode of delivery and gestational week at birth, gender of the newborn and time until the placenta was frozen were included as fixed effects in the model. No strong correlations were detected for these variables. EPDS scores measured at later gestational weeks had high correlation (Spearman rho 0.66 and p-value 3.14E-12 at 24 gwks, rho 0.61 and p-value 6.59E-10 at 34 gwks) with the score measured at gwk 14 and therefore were excluded from the model. Individual was included as a random effect. Sex chromosomes were removed from the analysis (979,084 sites left) and additional step was implemented to include information of the neighbouring CpG sites by using radmeth algorithm (adjust –bins 1:200:1) as previously described (Dolzhenko and Smith, 2014; Konki et al., 2020). Also Samtools v1.9 (Li et al., 2009) and R v3.6.2, v4.0.2 (Core_Team_(2013), 2013) computational environment was utilized in the analyses. The CpG sites in which we detected statistically significant association (FDR ≤ 0.05) with prenatal exposure to maternal depressive symptoms are also referred to as “DNA methylation marks” in this study.

### 2.4 Annotation and functional analyses of DNA methylation marks

Localization of the DNA methylation marks in genic and intergenic elements was performed by using Annotatr package v1.14.0 (Cavalcante and Sartor, 2017) and GREAT tool (McLean et al., 2010). To examine co-localization with histone marks, ChIP-seq data from 113 d placental samples for H3K27ac (ENCFF180ADH), H3K4me1 (ENCFF182QLP), H3K9me3 (ENCFF500BTY), H3K27me3 (ENCFF517ABG), H3K4me3 (ENCFF789GRZ) and H3K36me3 (ENCFF902ITG) provided by the NIH Roadmap Epigenomics project (http://www.roadmapepigenomics.org/) was utilized. Overlaps in genomic regions were identified with bedtools intersect function (Galaxy v2.29.2) (Quinlan and Hall, 2010). Enrichment was examined with hypergeometric distribution test (R phyper function) in comparison to the background measured with RRBS. Transcription factor binding and functional enrichment was examined with ggprofiler2 R package v0.2.0 (Raudvere et al., 2019). In addition, transcription factor binding sites were extracted from ENCODE data encRegTfbsClusteredWithCells.hg38.bed.gz (Davis et al., 2018; Dunham et al., 2012) available at UCSC (http://genome.ucsc.edu) (Karolchik et al., 2004). Tissue enrichment of the genes was examined with TissueEnrich v1.8.0 (Jain and Tuteja, 2019) and ABAEnrichment v1.18.0 (Grote et al., 2016) R packages. Enrichment of genetic variants from dbGaP and GWAS catalog was examined with traseR v1.18.0 (Chen and Qin, 2016). R studio (v4.0.2) (_4_0_0_R_D_C_T_2020), Jupyter Notebook v6.0.3 (Kluyver et al., 2016) computational environments and pandas v1.2.0, and seaborn v0.11.1 libraries were also utilized in the in depth analyses. Use of term enrichment or over-representation in the results section refers to statistically significant findings in these analyses after correction for multiple testing.

## 3 Results

### 3.1 Exposure to maternal depressive symptoms during early pregnancy is associated with DNA methylation marks in hundreds of genomic regions in placenta

The objective of our study was to determine whether exposure to maternal depressive symptoms measured at gwk 14 are associated with DNA methylation marks in placenta and, if such marks are identified, to gain insights into their potential functional significance. For this purpose, we analyzed DNA methylomes of 92 placentas, which had been collected from the deliveries by mothers with EPDS score ranging from 0 to 19. For statistical testing we used Penalized Quasi-Likelihood for sequencing count data package, which implements generalized linear mixed model. To test the associations, we set the null hypothesis *H*_*0*_ according to which DNA methylation of none of the measured CpG sites is associated with the maternal EPDS score measured during first trimester. According to our alternative hypothesis *H*_*A*_ DNA methylation of at least one or more CpG sites is associated with the EPDS score. Influence of covariates was controlled as described in the methods section.

**Fig. 1:**
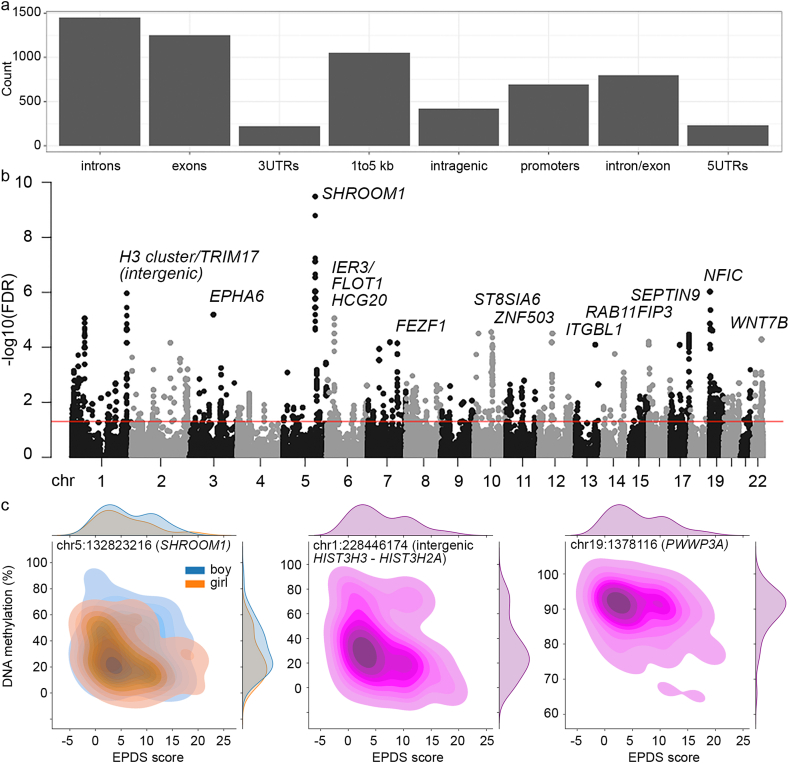
**The CpG site methylation associated with maternal depressive symptoms measured during early pregnancy.** DNA methylomes of placental samples were analyzed with reduced representation bisulfite sequencing. Association with maternal depressive symptoms (EPDS score at gestational week 14) was determined with generalized linear mixed model. A) distribution of the 2833 DNA methylation marks with FDR ≤0.05 in functional elements of the genome. B) distribution of the DNA methylation marks into the chromosomes and annotations of the overlapping or nearest genes with the highest false discovery rates (y-axis). C) Relationship of the DNA methylation levels and EPDS scores in different individuals for the three most significant sites presented with kernel density estimates. A full list of CpG sites (FDR ≤ 0.05) is available in Table B.

Our results supported the alternative hypothesis. The data revealed that DNA methylation levels of 2833 CpG sites are associated with the EPDS scores of the mothers with FDR ≤0.05 (Fig. 1 and Table B1). Of these sites approximately 62% localized in genic, 23% within 5 kb distance from transcription start site (TSS) of a gene and 15% in other intergenic regions. The sites were over-represented in exons (adj. pvalue 9.64E-12) and promoters (adj. pvalue 1.59E-29) and under-represented in intergenic regions (adj. pvalue 5.02E-20) and introns (adj. pvalue 1.17E-32) (Table B2). According to the annotatr tool the CpG sites were localized in the coding region or within 5 kb distance from the TSS of a total of 245 genes. GREAT tool found 375 additional genes. Merging of the regions within the same gene locus resulted in a total of 333 regions carrying DNA methylation marks.

**Table 1:** Enrichment of CpG sites associated with EPDS^a^ score in histone marks in placenta.

Histone mark detected in placenta	Overlapping measured CpG sites^b^ (no)	Overlapping measured CpG sites (%)	Overlapping CpGs associated with EPDS score (no)	Overlapping CpGs associated with EPDS score (%)	Enrichment (p-value)	Enrichment (BH adjusted p-value)
H3K4me1	232,067	24	1451	51	9.06E-221	5.32E-214
H3K27ac	93,273	10	820	29	2.69E-188	1.58E-181
H3K4me3	93,704	10	985	35	3.75E-294	2.20E-287
H3K36me3	39,060	4	119	4	2.64E-01	1.55E+06
H3K27me3	190,387	19	553	20	4.49E-01	2.64E+06
H3K9me3	73,506	8	224	8	1.99E-01	1.17E+06

a Edinburgh postnatal depression scale.

b Measured refers to the CpG sites measured with reduced representation bisulfite sequencing and used as a background set in the hypergeometric test.

**Table 2:** Enrichment of variants with known clinical significance in the genomic regions harboring DNA methylation marks including loci in linkage disequilibrium.

Trait	SNP^a^ hits	total SNPs	p value	q value	odds ratio
Attention deficit disorder with hyperactivity	5	461	2.21E-09	1.26E-06	124.11
Monocyte chemoattractant protein 1 (66–77)	3	444	1.67E-05	4.78E-03	85.55
Bipolar disorder	3	709	6.65E-05	1.27E-02	53.48
Mental disorders (trait class)	8	3725	1.02E-08	3.36E-07	22.88

a Single nucleotide polymorphism.

For 81% of the CpG sites the beta coefficients were negative after adjustment for other covariates. This indicates that increase in the EPDS score has negative influence on DNA methylation of most of the CpG sites. The eleven most significant DNA methylation marks were detected in the genic regions of eight genes, including *SHROOM1*, *PWWP3A, NFIC, EPHA6, LRP8, IER3*, *DOCK6* and *ST8SIA6* and in three intergenic regions. The intergenic regions were located between *HIST3H3* and *HIST3H2A* genes, *NODAL* and *PALD1* genes, and *RSP26* and *ERBB3* genes. The density distribution of DNA methylation versus EPDS score for the three top sites is shown in Fig. 1c. No significant sites were detected in the placental genes *NR3C1* or *HSD11B2*, which have been associated with the prenatal exposure to maternal depressive symptoms in previous targeted analyses (Conradt et al., 2013; Monk et al., 2016; Stroud et al., 2016). In the *HSD11B2* gene two nominally significant sites, chr16:67431674 (p-value 5.60E-03) and 16:67431658 (p-value 1.31E-02), were detected, however, these became insignificant after correction for multiple testing. The most significant DNA methylation mark in *SHROOM1* gene was also influenced by gender, *EPHA6* gene was influenced by maternal smoking, *NFIC* was influenced by gender, gestational age and labor mode and *LRP8* was influenced by maternal gestational diabetes, BMI, gestational age and maternal age (FDR ≤ 0.05). Altogether the covariates influenced DNA methylation of 1309 sites or 139 regions, which were associated with the maternal EPDS score (Table B3).

**Table 3:** Enrichment of transcription factors with binding sites in DNA methylation marks in extracellular signal mediated pathways (see full list of terms in Table E).

Term name	Adjusted p value
ESR-mediated signaling	5.12E-18
TGF-beta Signaling Pathway	1.65E-07
Androgen receptor signaling pathway	5.44E-07
Signaling by NTRK1 (TRKA)	8.40E-07
TGF-beta Receptor Signaling	7.68E-06
Thyroid hormone signaling pathway	5.13E-05
Parathyroid hormone synthesis, secretion and action	8.11E-05
Signaling by NOTCH	4.99E-04
PDGFR-beta pathway	2.24E-03
IL-4 Signaling Pathway	3.34E-03
TNF signaling pathway	5.60E-03
Estrogen signaling pathway	5.81E-03
Aryl Hydrocarbon Receptor Pathway	7.02E-03
Growth hormone synthesis, secretion and action	8.73E-03
IL-5 Signaling Pathway	2.03E-02
Wnt signaling pathway	2.54E-02
Serotonin Receptor 4/6/7 and NR3C Signaling	2.54E-02
IL-6 signaling pathway	3.26E-02
EGF/EGFR Signaling Pathway	3.91E-02

Based on these results we rejected our null hypothesis and concluded that maternal depressive symptoms during early pregnancy are associated with DNA methylation marks in hundreds of genomic regions in the placenta.

### 3.2 Overlap of the DNA methylation marks with the functional elements of the genome

**Fig. 2:**
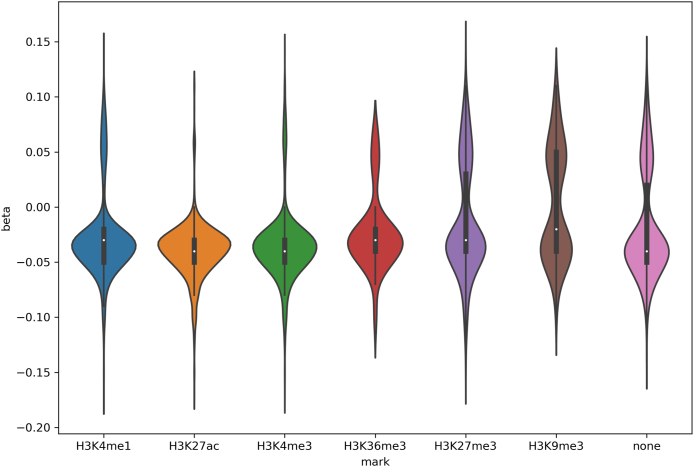
**The beta values of the CpG sites co-localized with histone marks in placental tissue.** DNA methylomes of placental samples were analyzed with reduced representation bisulfite sequencing. Association of CpG methylation with maternal depressive symptoms (EPDS score at gestational week 14) was determined with generalized linear mixed model. Genomic co-localization of the DNA methylation marks (FDR ≤ 0.05) with histone marks previously detected in placental tissue was determined. The figure represents the beta values of the CpG sites associated with maternal depressive symptoms (y-axis). The CpG sites are grouped based on their colocalization with histone marks (x-axis). A full list of CpG sites with beta values (FDR ≤ 0.05) is available in Table B.

To further elucidate, how the DNA methylation marks associated with exposure to maternal depressive symptoms may influence gene function in placenta, we studied co-localization of the regions with the functional elements of the genome carrying histone marks. More specifically, we tested over-representation of the affected regions in the enhancer marks H3K4me1 and H3K27ac, promoter mark H3K4me3, elongation mark H3K36me3, Polycomb repressive mark H3K27me3 and heterochromatin mark H3K9me3 that have been detected in the human placenta at day 113. Of the 2833 sites, 51% were found to overlap with an enhancer mark H3K4me1 in placenta (Table 1). Enrichment of the affected sites in these enhancers was statistically significant (BH adjusted p-value 9.06E-221). Interestingly, 84% of these sites overlapping with enhancers were localized in the intragenic regions or within 5 kb distance from a transcription start site. No over-representation of the DNA methylation marks was detected in repressive histone marks or elongation mark. For the H3K4me1, H3K27ac and H3K4me3 marks the beta values were mostly negative. For example, 89% CpG sites overlapping H3K4me1 enhancer mark and 94% overlapping H3K4me3 promoter mark had a negative beta value. Also, for negative marks the more negative beta values were detected, however, the proportion of positive beta values was higher for these sites (Fig. 2). For example, 44% of the CpG sites carrying heterochromatin mark H3K9me3 and 31% carrying H3K27me3 mark had positive beta values.

As a conclusion, maternal depressive symptoms during early pregnancy are associated with DNA methylation status of specific genomic regions, which are mostly localized in genic and promoter regions. The affected regions are particularly enriched in the genic enhancers where maternal depressive symptoms are mostly associated with decreased rather than increased DNA methylation.

### 3.3 The DNA methylation marks are localized in genes involved in development of neurons

To determine, whether the DNA methylation marks associated with maternal depressive symptoms are enriched for any known biological processes or phenotypes, we performed over-representation analysis of these genomic sites with gprofiler2. Statistically significant results were found for the terms neuron differentiation (adj.pval 1.28 × 10^-2), generation of neurons (adj.pval 2.3 × 10^-2), neurogenesis (adj.pval 2.45 × 10^-2) and regulation of cell communication (adj.pval 4.98 × 10^-2). Over-representation of 99 transcription factor binding motifs were also found in these regions (adj.pval ≤ 5.00 × 10^-2, Table C1). For the genes harboring the regions with DNA methylation marks, similar and additional functional enrichments in more general developmental processes were found (Table C2).

As a conclusion, maternal depressive symptoms during early pregnancy are associated with DNA methylation marks, which are enriched in genes involved in development of neurons.

### 3.4 Expression of genes harboring DNA marks is enriched in several regions of developing brain

We then asked whether the expression of the genes harboring DNA methylation marks is over-represented in any specific tissue, such as placenta. To address this question, we used TissueEnrich package. A total of 245 nearest genes identified by annotatr and additional 375 nearest genes identified by great tool were included in analysis. Expression of these genes was examined in 35 tissues available from Human Protein Atlas, including placenta, and also in GTEx dataset. Based on the results no statistically significant enrichment of the genes harboring DNA methylation marks into any of the tissues or tissue group was detected in the data.

As functional enrichment analysis suggested that the genes harboring the DNA methylation marks may be important for generation and development of neurons, we further examined expression of these genes more specifically in data from different regions of brain during development and in adults. For this purpose we used data from Allen Brain Atlas project and BrainSpan Atlas of the Developing Human Brain and ABAEnrichment R package. According to the results, expression of the genes in developmental data set was over-represented in several brain regions in particular in data from prenatal and infant brain. Statistically significant enrichments (min FWER 5.00E-02) were found in eight regions in prenatal, twelve in infant, two in childhood, three in adolescent and one in adult brain data. For example, over-representation was detected at several developmental stages in amygdaloid complex, from prenatal (min FWER 4.00E-02), infant (min FWER 2.10E-02) and childhood (min FWER 3.90E-02) brain data and cerebellar cortex from prenatal (min FWER 2.00E-03), infant (min FWER 7.00E-03) and adolescent (min FWER 1.90E-02) brain data. Enrichments in ventrolateral prefrontal cortex, frontal neocortex and dorsolateral temporal neocortex (min FWER < 5.00E-02) were detected in both prenatal and infant data, and striatum in both infant (min FWER 0) and childhood (min FWER 3.60E-02) data. In adolescent brain data the most significant over-representation was detected in hippocampus (min FWER 1.00E-03) and in adult brain data in ICjl_interstitial nucleus of Cajal (right) (min FWER 3.80E-02). The complete list of results is provided in Table D.

As a conclusion, expression of the genes, which overlap with the DNA methylation marks for exposure to maternal depressive symptoms, is enriched in several brain regions in particular in data collected from prenatal and infant brain (Grote et al., 2016). Enrichments at several developmental stages were detected in amygdaloid complex and cerebellar cortex.

### 3.5 Genetic variants enriched in the genomic regions harboring DNA methylation marks

To elucidate potential functional importance of the genomic regions with DNA methylation marks for different traits and disorders, we examined over-representation of genetic variants in these regions by using traseR R package, which is synchronized with dbGaP and GWAS catalogue. The test was performed for a total of 573 traits, 33 trait classes and 78,247 SNPs available in the databases. According to the results the genomic regions harboring DNA methylation marks were statistically significantly enriched (q-value ≤ 5.00E-02) for alleles in linkage disequilibrium with single nucleotide polymorphisms, which have been previously associated with attention deficit disorder with hyperactivity (ADHD), monocyte chemoattractant protein 1 (MCP1) level, bipolar disorder and in the mental disorder trait class (Table 2).

As a conclusion, genomic regions harboring DNA methylation marks for prenatal exposure to maternal depressive symptoms are enriched for variants previously associated with mental disorders.

### 3.6 Transcription factors with binding sites in the DNA methylation marks are enriched in signaling pathways with potential to mediate effects of prenatal exposure to maternal depressive symptoms

In order to gain insights into the potential upstream regulators of the DNA methylation marks, we merged the CpG sites within 25 bp distance from each other, extracted transcription factors binding into these regions based on data available from ENCODE (Davis et al., 2018; Dunham et al., 2012) and performed over-representation analysis with gprofiler. Numerous statistically significant biological processes and phenotypes were identified (adjusted p-value ≤ 0.05, Table E). From these we collected the signaling pathways mediated by extracellular molecules. A total of 17 non-redundant signaling pathways were found (Table 3). Six of these were mediated by hormones estrogen, androgen, thyroid hormone, parathyroid hormone, growth hormone and serotonin. Five were mediated by cytokines, including TGFB, IL4, TNF, IL5 and IL6, three by growth factors TRKA, EGF and PDGF. In addition, developmentally important WNT and NOTCH signaling and Aryl Hydrocarbon Receptor Pathway were also among the significant pathways. A full list of terms associated with the transcription factors can be found in Table E. In conclusion, multiple signaling pathways with potential to mediate the influence of prenatal exposure to maternal depressive symptoms on placental DNA methylation were identified.

## 4 Discussion

To our knowledge we show for the first time that maternal depressive symptoms measured during early pregnancy are associated with DNA methylation levels of thousands of CpG sites in the placenta. Most of these sites were found in the gene bodies or promoters. We also identified several cellular signaling pathways, such as cytokine and hormonal cascades, which may regulate these genomic regions. These DNA methylation marks and pathways provide valuable candidates to be verified in the future studies. The most significant DNA methylation marks were found in the intron of *SHROOM1* gene, exon of *PWWP3A* gene and in the intergenic region between *HIST3H3* and *HIST3H2A* genes. The beta values in *SHROOM1* gene were also influenced by gender. The functional significance of these genes in placenta is unclear. The protein encoded by the *SHROOM1* gene is a cellular architecture protein, which in a mouse model, has been observed to be required for appropriate neurulation (Hildebrand and Soriano, 1999). PWWP3A protein has been reported to promote cell survival by inducing chromatin compaction in response to genotoxic stress (Huen et al., 2010), whereas, histone 3 is the core component of the nucleosome with crucial function in maintenance of chromatin organization and function. In addition to these top three regions, association was found in hundreds of other genomic regions, however, not in the genes *NR3C1* and *HSD11B2,* which have been previously associated with prenatal exposure to maternal depressive symptoms (Conradt et al., 2013; Monk et al., 2016; Stroud et al., 2016). Nevertheless, our findings do not rule out existence of such marks, as comparison to previous findings is challenged by the differences in statistical approaches and methods used to produce the results. Furthermore, in our study we have not necessarily covered exactly the same CpG sites, which have been measured with targeted approaches.

Although we identified hundreds of new DNA methylation marks, in this study we did not measure their potential influence on gene expression. Few previous studies exist, which have used targeted approaches to compare gene expression differences of placentas from mothers with and without depression. For example, Edvinsson et al. found nominally significant higher expression of *HTR1A* and *NPY2R* genes in the placentas from untreated depressed mothers and higher HTR1A protein level in the placentas of mothers on antidepressant treatment in comparison to healthy controls (Edvinsson et al., 2019). We did not find DNA methylation marks in the proximity of these genes. Litzky et al. compared differences in the expression of imprinted genes in placentas from mothers with history or antenatal depression and/or anxiety (Litzky et al., 2018). Consistently with their findings, we found DNA methylation marks near the gene *ERLIN2*. According to our results, the DNA methylation marks are over-represented in the intragenic enhancers and currently it is unclear how DNA methylation in these genomic elements influences gene activity. Previous studies have indicated that intragenic enhancers are mostly localized in transcriptionally active genes and may be important in activation, attenuation and fine-tuning expression of their host gene (Cinghu et al., 2017). Therefore, one may speculate that the genes we have identified are likely to be active in placenta and exposure to maternal depressive symptoms may alter their activity. However, perhaps unexpectedly, our in silico analyses did not find any significant functions or overexpression of these genes in placenta. Instead, our results suggest that the genes nearest to the DNA methylation marks are important regulators of neuronal and brain development and expression of these genes is enriched in several regions of developing brain, including amygdaloid complex in infants and hippocampus in adolescents. Based on these findings one may hypothesize that similarly to the placenta, the developing fetus has been exposed to the same epigenetic modifiers targeting the same genomic regions and genes with tissue specific influence on brain development rather than placental function. This hypothesis is also supported by findings from our previous study in which we found weak statistical evidence for interaction between offspring polygenic risk score for major depressive symptoms and prenatal maternal depressive symptoms on right amygdalar volumes in infants participating in the FinnBrain Cohort Study (Acosta et al., 2020). In addition, a sex-specific gene x environment effect on right hippocampal volumes was found (Acosta et al., 2020). Also several other studies have found association between prenatal exposure to maternal depressive symptoms and brain imaging-derived and behavioral phenotypes (Hay et al., 2020; A. Lee et al., 2019; Qiu et al., 2015; Rifkin-Graboi et al., 2013; Soe et al., 2018; van der Knaap et al., 2018; Wen et al., 2017). For example, in girls gender specific associations have been found between prenatal exposure to maternal depressive symptoms and larger right amygdala volumes at 4.5 years of age (Wen et al., 2017) and in boys weaker connectivity in the amygdala-frontal pathway and worse externalizing behavior has been detected at the age of 2.9–6.0 years (Hay et al., 2020). Whether these phenotypes are mediated through epigenetic mechanisms remains unclear. Due to limited access to brain tissue, epigenome-wide association analysis of these brain regions is perhaps not feasible. Therefore, further follow-up studies identifying DNA methylation marks associated with the phenotypic outcomes and detected non-invasively in tissues, such as placenta, will be highly valuable.

As trait-associated single nucleotide variants have potential to indicate whether a genomic region of interest is likely to be functionally linked to a particular phenotype or disease outcome (Chen and Qin, 2016), we examined over-representation of such variants in those genomic regions in which we found DNA methylation marks. Our results revealed enrichment of variants associated with psychiatric disorders, including ADHD and bipolar disorder in these regions, suggesting that the DNA methylation marks we have identified may be functionally linked to these disorders. This is perhaps not surprising as previous studies have associated maternal prenatal depression with ADHD symptoms of the child and substantial proportion of this association was explained by shared genetic background (Eilertsen et al., 2020). Furthermore, genome-wide association studies have found that major depressive disorder, ADHD and bipolar disorder share common genetic risk variants (Anttila et al., 2018; P. H. Lee et al., 2019). Previous studies on placenta (Chatterjee et al., 2021) and umbilical cord blood (Teh et al., 2014) have also found that interindividual variation in DNA methylation is most often explained by gene-environment interaction and to less extent by genetic factors whereas environmental factors alone rarely explain the variation. Therefore, based on this knowledge it is likely that most of the DNA methylation marks we have detected are co-influenced by genetic variants. Whether these placental marks are associated with the developmental and health outcomes of the corresponding children, as has been previously reported for *NR3C1* methylation (Conradt et al., 2013), remains to be elucidated in the future studies.

In conclusion, our results suggest that maternal depressive symptoms during early pregnancy are associated with DNA methylation marks in hundreds of genomic regions in placenta. The marks are enriched in genes, which are important for the development and long-term health of the brain. It remains to be elucidated in further studies whether similar marks can be detected in the exposed children and whether these marks are prognostic for the developmental and health outcomes. Further studies are also needed to determine the potential impact of the detected DNA methylation marks on gene regulation and cellular functions.

Limitations of our study were that we did not control for the birth weight and the DNA methylome analysis was performed by using heterogenous bulk tissue as a starting material. In addition, we used reduced representation bisulfite sequencing for DNA methylome analysis. Therefore, our data does not cover the whole genome, which hampers comparison of the results to previous targeted studies.

## Author contributions

HK and LK established and administered the FinnBrain Birth Cohort Study and provided expertise support for the study. EE, NP, LK, NS, RLu planned the experimental design. EE supervised collection of the placental tissues and clinical characterization of the samples. NP collected the sample characteristics. RK isolated the genomic DNAs, MKo prepared RRBS libraries, MKy performed processing of the raw data and bioinformatic analyses with PQLSeq. RLu supervised the laboratory work, processing of raw data and PQLSeq analyses, performed in depth bioinformatic analyses, interpretation of the results and drafted and finalized the manuscript. HK, LK, EE, NS and MK contributed to the scientific content of the manuscript. All authors had an opportunity to review the manuscript.

## Declaration of competing interest

Declarations of interest: none.
